# Prediction of cognitive performance by demographics, sleep, and brain morphometry: machine learning findings from ENIGMA-Sleep Working Group

**DOI:** 10.21203/rs.3.rs-9914920/v1

**Published:** 2026-06-09

**Authors:** Hanwen Bi, Torbjörn Åkerstedt, Robin Bülow, Michele Deantoni, Alexander Drzezga, Jeremy A. Elman, David Elmenhorst, Eva-Maria Elmenhorst, Ralf Ewert, Christine Fennema-Notestine, Fabio Ferrarelli, Stefan Frenzel, Charlotte von Gall, Sarah Genon, Hans J. Grabe, Phuong Thuy Nguyen Ho, Sanne J.W. Hoepel, Felix Hoffstaedter, Agustin Ibanez, Neda Jahanshad, Ahmadreza Keihani, Vincent Küppers, Wen Liu, Ahmad Mayeli, Nasrin Mortazavi, Julia Neitzel, Gustav Nilsonne, Matthew S. Panizzon, Julia S. Rupp, Amin Saberi, Christina Schmidt, Kai Spiegelhalder, Beate Stubbe, Sandra Tamm, Sophia I. Thomopoulos, Paul M. Thompson, Sofie L. Valk, Gilles Vandewalle, Tina Thi Vo-Eckerle, Henry Völzke, Laura K. Waite, Joseph Wexler, Katharina Wittfeld, Kaustubh R. Patil, Antoine Weihs, Simon B. Eickhoff, Federico Raimondo, Masoud Tahmasian, ENIGMA-Sleep Working Group

**Affiliations:** 1 Institute of Neuroscience and Medicine, Brain and Behaviour (INM-7), Research Center Jülich, Jülich, Germany; 2 Institute of Systems Neuroscience, Medical Faculty and University Hospital Düsseldorf, Heinrich Heine University, Düsseldorf, Germany; 3 Department of Clinical Neuroscience, Karolinska Institutet, Stockholm, Sweden; 4 Department of Psychology, Stockholm University, Stockholm, Sweden; 5 Institute of Diagnostic Radiology and Neuroradiology, University Medicine Greifswald, Greifswald, Germany; 6 GIGA-CRC-Human Imaging, University of Liège, Liège, Belgium; 7 Department of Nuclear Medicine, Faculty of Medicine and University Hospital Cologne, University of Cologne, Cologne, Germany; 8 German Center for Neurodegenerative Diseases (DZNE), Bonn-Cologne, Germany; 9 Institute for Neuroscience and Medicine, Molecular Organization of the Brain (INM-2), Research Center Jülich, Jülich, Germany; 10 Center for Behavior Genetics of Aging, Department of Psychiatry, University of California San Diego; 11 Department of Psychiatry, University of California San Diego; 12 Department of Sleep and Human Factors Research, German Aerospace Center, Cologne, Germany; 13 Institute for Occupational, Social and Environmental Medicine, Medical Faculty, RWTH Aachen University, Aachen, Germany; 14 Department of Internal Medicine B-Cardiology, Pneumology, Infectious Diseases, Intensive Care Medicine, University Medicine Greifswald; 15 Department of Psychiatry, University of Pittsburgh, Pittsburgh, PA, USA; 16 Department of Psychiatry and Psychotherapy, University Medicine Greifswald, Greifswald, Germany; 17 Institute of Anatomy II, Medical Faculty, Heinrich Heine University, Düsseldorf, Germany; 18 German Center for Neurodegenerative Diseases (DZNE), Site Rostock/Greifswald, Greifswald, Germany; 19 Department of Radiology and Nuclear Medicine, Erasmus University Medical Centre, Rotterdam, the Netherlands; 20 Department of Epidemiology, Erasmus MC University Medical Center Rotterdam, Rotterdam, Netherlands; 21 Latin American Brain Health Institute (BrainLat), Universidad Adolfo Ibáñez, Santiago, Chile; 22 Global Brain Health Institute, Trinity College Dublin, Dublin, Ireland; 23 Department of Biophysics, School of Medicine, Istanbul Medipol University, 34815 Istanbul, Türkiye; 24 Barcelonaβeta Brain Research Center (BBRC), Pasqual Maragall Foundation, 08005, Barcelona, Spain; 25 Cognitive Neuroscience Center (CNC), Universidad de San Andrés, Buenos Aires, Argentina; 26 Imaging Genetics Center, Mark and Mary Stevens Neuroimaging and Informatics Institute, Keck School of Medicine of the University of Southern California, Los Angeles, CA, USA; 27 Department of Epidemiology, Harvard T. H. Chan School of Public Health, Boston, Massachusetts; 28 International Globally Distributed Organization for Research and Education (IGDORE), Stockholm, Sweden; 29 Max Planck Institute for Human Cognitive and Brain Sciences, Leipzig, Germany; 30 Department of Psychiatry and Psychotherapy, Medical Center – University of Freiburg, Faculty of Medicine, University of Freiburg, Germany; 31 Institute for Community Medicine, SHIP/Clinical-Epidemiological Research, University Medicine Greifswald, Greifswald, Germany; 32 German Centre for Cardiovascular Research (DZHK), Partner Site Greifswald, Greifswald, Germany; 33 Department of Psychology, Stanford University, Stanford, CA, USA

## Abstract

Group-level studies have highlighted the roles of aging, poor sleep, and brain atrophy in cognitive performance (CP) but have overlooked inter-individual variability. We predict CP from feature sets (demographic, subjective/objective sleep parameters, and regional brain morphometry) using multisite ENIGMA-Sleep data (n = 2,372). Linear and non-linear machine learning models were trained on the largest cohort (n = 845), and the best-performing models were validated on independent cohorts. Subsequently, based on the best-performing model on the largest cohort, we characterized feature importance and interactions across all cohorts. We observed that a combination of demographic, sleep, and brain parameters moderately predicted CP, with age emerging as the key predictor. Model explanations further suggested that age was the primary driver of prediction models, while sleep played a smaller role that varied across subgroups. These findings endorsed inter-individual variability and complex interaction between aging, sleep, brain, and CP.

## INTRODUCTION

Cognitive performance (CP) reflects individual ability across core neurocognitive domains, including attention, processing speed, executive function, and memory. In population studies, lower CP is associated with poorer educational and occupational attainment, reduced everyday functioning, maladaptive interpersonal relationships, and adverse mental health outcomes across the lifespan (Lövdén et al., 2020). Thus, poor CP, including attention and memory impairments, can lead to substantial healthcare and societal costs (Gläser et al., 2025). Notably, substantial inter-individual variability in CP exists, influenced by biological, lifestyle, and environmental factors. Identifying which factors most strongly contribute to CP at the individual level is therefore critical for informing early intervention strategies and guiding personalized approaches to cognitive health.

Aging across the lifespan is associated with cognitive decline in multiple domains and is an early symptom for the development of prevalent neurodegenerative diseases such as dementia (Brito et al., 2023). Fluid cognitive abilities, including attention, novel problem-solving, processing speed, literacy, numeracy, and episodic memory, tend to decline gradually from midlife onward, particularly in women (Brito et al., 2023; Hanushek et al., 2025). Accordingly, aging poses a major challenge to maintaining CP in rapidly aging populations. Besides aging, sleep is related to multiple aspects of CP (Tahmasian et al., 2026). Previous large-scale studies in a large aging population dataset (UK Biobank, UKB) observed that both long and short sleep durations were linked to impaired CP (Kyle et al., 2017; Li et al., 2025), and multi-organ ageing clocks (O’Toole et al., 2026). Non-linear relationships between sleep duration and CP and executive function have also been reported (Li et al., 2022; Tai et al., 2022). Longitudinal studies have shown that abnormal sleep duration and shift work are predictors of future cognitive impairment (Ell et al., 2023). Although these studies reported statistically significant group-level associations, the corresponding effect sizes were consistently very small (r range 0.0007 - 0.0057) (Keyes & Westreich, 2019; Lin et al., 2013; Spence et al., 2026; Sullivan & Feinn, 2012). Those UKB studies also primarily relied on a single self-reported question about sleep duration from the British population, whereas sleep behaviors show substantial regional and cultural variation (Coutrot et al., 2022; Willoughby et al., 2023). Moreover, individual sleep needs are flexible across individuals and dynamically related to CP (Fjell & Walhovd, 2024). Together, these factors may limit the generalizability of group-level findings to international cohorts.

The neurobiological substrates linking CP, aging, and sleep remain poorly understood, making the characterization of sleep-related variation in brain structure essential for elucidating the mechanisms underlying their interplay. Prior work incorporating genetic information has reported non-linear relationships among sleep duration, cognition, and brain structure (Li et al., 2022), with divergent patterns between short- and long- sleep duration groups (Li et al., 2025). An inverse U-shaped cross-sectional association between sleep duration and cortical thickness was reported, but longitudinal analysis did not show robust associations (Fjell, Sørensen, Wang, Amlien, Baaré, Bartrés-Faz, Bertram, et al., 2023). Other evidence suggests that long sleep duration is linked with higher grey matter volume of the basal ganglia, whereas insomnia symptoms were not associated with morphological measurements (Schiel et al., 2023; Weihs et al., 2023). At the individual level, structural brain data have been used to predict CP and sleep health separately, suggesting that morphometric measures may capture variability relevant to CP, although their predictive power has generally been modest (Genon et al., 2022; Raimondo et al., 2026; Wulan et al., 2024). Taken together, these findings suggest inconsistencies across studies regarding the interplay between aging, sleep, brain, and CP. Large-scale international datasets and advanced computational approaches are therefore needed to model this complexity and clarify the cognitive consequences of poor sleep, aging, and brain atrophy.

Machine learning (ML) provides an individual-level framework that can accommodate heterogeneous, non-linear associations, and particularly in unseen data (independent cohorts), thereby improving generalizability beyond traditional group-level statistical analyses in one cohort (Varoquaux & Poldrack 2019). In this preregistered study (https://osf.io/nhbkq/), we leveraged multisite international data of the ENIGMA-Sleep Working group (Tahmasian et al., 2021) with both self-reported and objective (polysomnography-based) sleep measures to evaluate whether demographics (i.e., age, sex, body mass index (BMI), and depressive symptom scores), sleep, and regional (parcel-based) brain morphometry can predict CP in the general population. To assess the contribution of single and multiple domains to predicting CP, we systematically compared ML models based on demographics-only, sleep with demographics, and a combination of demographics, sleep, and brain morphometry, and complemented these comparisons with shuffled-feature controls. To evaluate the generalizability of ML models beyond a single cohort and under geographic variation in sleep behaviors (Coutrot et al., 2022; Willoughby et al., 2023), we trained multiple ML models in the largest cohort and assessed the best-performing models on independent cohorts, consistent with recommendations that external validation provides a more rigorous assessment of generalizability in neuroimaging prediction studies (Bi et al., 2026; Olfati et al., 2024; Rosenblatt et al., 2026). We then applied explainable ML using SHapley Additive exPlanations (SHAP) (Lundberg & Lee, 2017) to identify feature contributions and also Shapley Interaction Quantification (SHAP-IQ) (Muschalik et al., 2024) to characterize feature interaction patterns underlying model predictions. To examine whether these prediction patterns were homogeneous across participants, we additionally clustered participant-specific SHAP values and explored the transferability of the resulting clustering rules across cohorts.

## RESULTS

### Overview of the framework and study cohort

**Figure 1.**
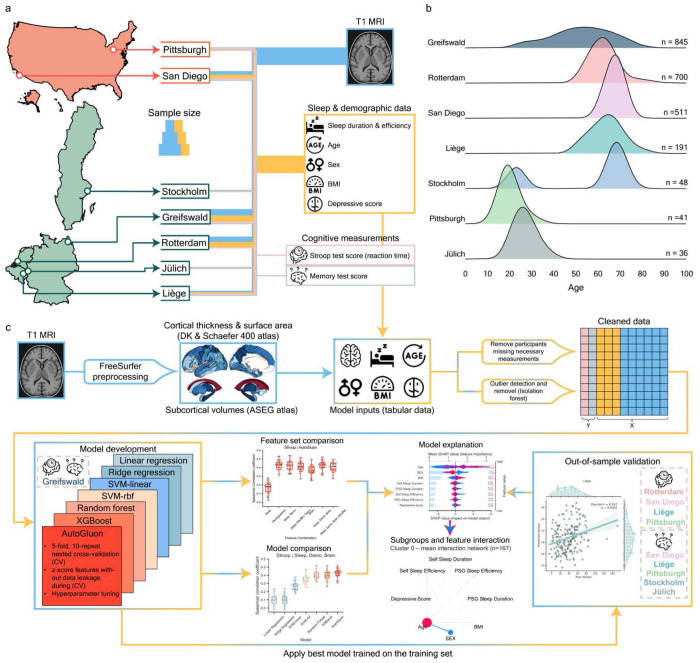
Data description and machine learning pipeline. **a,** Data were collected from 2,372 participants across seven cohorts within the ENIGMA-Sleep working group. The datasets included demographic information, sleep measurements, cognitive performance scores, and structural brain imaging data derived from T1-weighted MRI scans. **b,** Cohorts differed in age distributions, ranging from young-adult to predominantly older-adult samples. Greifswald cohort spans early to late adulthood and was used for model development. Details for each cohort are provided in Supplementary Figure 1 and Supplementary Table 1. **c,** T1 MRI preprocessing was performed with FreeSurfer’s recon-all command to derive parcel-based cortical and subcortical measures. Data were integrated into a tabular format and exclusion of participants with incomplete data and outlier removal. Models were trained in the Greifswald cohort with nested crossvalidation. Validation of Stroop predictions was conducted using data from Rotterdam, San Diego, Liège, and Pittsburgh, while validation of memory predictions utilized data from San Diego, Liège, Stockholm, Pittsburgh, and Jülich. Model explanations were generated with SHAP and SHAP-IQ to quantify feature importance and interactions. Participant subgroups were identified by spectral clustering based on SHAP values.

We evaluated whether demographics, sleep, and regional brain morphometry features could predict CP across seven international cohorts (n = 2,372 participants), including Greifswald (SHIP-Trend, n = 845) (Völzke et al., 2022), Rotterdam (The Rotterdam Study, n = 700) (Ikram et al., 2024), San Diego (VETSA, n = 511) (Kremen et al., 2013), Liège (COF and COGNAP, n = 191) (Deantoni et al., 2024), Stockholm (Sleepy Brain, n = 48) (Nilsonne et al., 2020), Pittsburgh (PyNEL, n = 41) (LaGoy et al., 2022), and Jülich (Somnosafe, n = 36) (Hennecke et al., 2021). The models used three feature domains, which were *i)* demographics including age, sex, BMI, and depressive scores, *ii)* sleep duration and efficiency derived from polysomnography (PSG) and self-reported questionnaires, and *iii)* parcel-based brain structural measures from T1 MRI, including cortical thickness, surface area, and subcortical volume. The prediction targets were cognitive measurements assessed by the Stroop (reaction time) and memory tests (accuracy). To facilitate cross-cohort interpretation, we defined the Stroop test score uniformly as reaction time and the memory test score as accuracy hereinafter. We trained the ML models in the Greifswald cohort, which included 845 participants and provided the largest sample size, the broadest age range, and both cognitive outcomes. Given differences in cognitive measurements and score scales across cohorts (Supplementary Figure 1), Spearman’s rank correlation coefficient was used as the primary performance metric to assess whether participants with better observed cognitive performance also tended to receive better predicted scores, without requiring exact agreement in score units. We then assessed the generalizability of best-performing ML models in independent cohorts using out-of-cohort validation. We first benchmarked seven linear and non-linear ML algorithms, then compared feature combinations to assess whether sleep measurements and parcel-based brain morphometry improved prediction beyond demographics. Based on the selected models, we used SHapley Additive exPlanations (SHAP) (Lundberg & Lee, 2017) and Shapley Interaction Quantification (SHAP-IQ) (Muschalik et al., 2024) to characterize feature importance and their interactions. To assess whether these explanation patterns were homogeneous across participants or instead reflected distinct participant profiles, we further applied spectral clustering to SHAP values in the Greifswald cohort and evaluated the transferability of the resulting clustering rules in the remaining cohorts (Fig. 1c).

### Prediction of cognitive performance in the Greifswald cohort

We first compared models based on demographics, sleep, brain morphometry features, and combinations to predict the Stroop and memory test scores in the Greifswald cohort using 5-fold 10-repeat nested crossvalidation. Across both outcomes, the demographics-only model showed the strongest performance among single-domain feature sets, whereas sleep-only and brain-only models performed worse. Adding sleep to demographics or adding sleep and parcel-based brain morphometry to demographics did not significantly improve prediction beyond the demographics-only model. Non-linear models showed the most robust performance when the complete feature set was used. Therefore, AutoGluon was used as the baseline model for feature-set comparisons because it matched the best linear models with demographics-only and outperformed them with the complete feature set (Supplementary Figure 2).

#### Stroop test:

**Figure 2.**
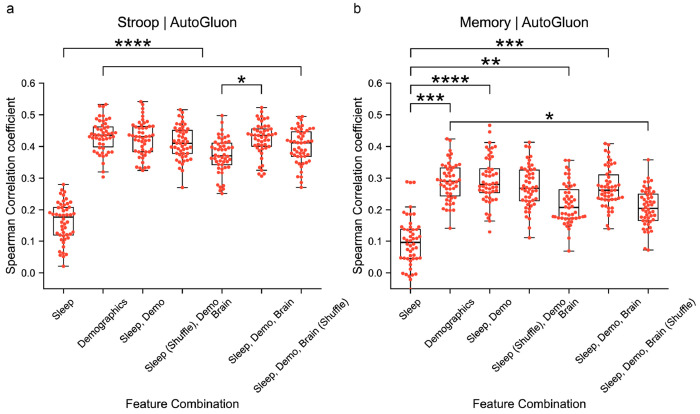
Prediction of cognitive performance by feature combinations in the Greifswald cohort using AutoGluon. **a,** Stroop test score prediction across feature combinations by AutoGluon. Boxplots display test Spearman’s rank correlation coefficient from 5-fold 10-repeat nested CV for feature combinations including sleep measurements only, demographics only, sleep with demographics, parcel-based brain morphometry only, and sleep, demographics with brain. Shuffled-feature controls for sleep measurements and parcel-based brain morphometry. **b,** Memory score prediction across the same feature combinations and evaluation scheme. Pairwise model comparison used Nadeau–Bengio corrected resampled paired t-tests on nested cross-validation scores with Bonferroni adjustment. Statistical significance: * P < 0.05, ** P < 0.01, *** P < 0.001, **** P < 0.0001.

With demographics-only features, AutoGluon achieved a robust prediction performance (test Spearman’s ρ = 0.43 ± 0.05, R^2^ = 0.15 ± 0.05, RMSE = 10.36 ± 1.48). Compared with the demographics-only model, the sleep-only model (test ρ = 0.16 ± 0.06) performed worse (P < 0.0001), and the brain-only model yielded intermediate performance (test ρ = 0.37 ± 0.06, R^2^ = 0.11 ± 0.04, RMSE = 10.60 ± 1.49). Adding sleep to demographics (test ρ = 0.43 ± 0.05, R^2^ = 0.15 ± 0.04, RMSE = 10.34 ± 1.49) matched the demographics-only model and using all features together (test ρ = 0.42 ± 0.05, R^2^ = 0.14 ± 0.04, RMSE = 10.42 ± 1.52), which was not significantly different from the demographics-only model. Shuffled-feature controls for sleep in sleep with demographics, and for brain in all feature sets, reduced performance slightly but not significantly (Fig. 2a, Supplementary Table 3).

#### Memory test:

A similar pattern was observed for memory test score prediction. With demographics-only features, AutoGluon showed a robust prediction performance (test ρ = 0.29 ± 0.06, R^2^ = 0.08 ± 0.04, RMSE = 15.08 ± 0.67). Compared with demographics-only, the sleep-only model (test ρ = 0.10 ± 0.08) performed worse (P < 0.001), and the brain-only model yielded intermediate performance (test ρ = 0.22 ± 0.06, R^2^ = 0.04 ± 0.02, RMSE = 15.42 ± 0.63). Adding sleep to demographics (test ρ = 0.29 ± 0.06, R^2^ = 0.08 ± 0.04, RMSE = 15.06 ± 0.69) matched the demographics-only model and using all features together (test ρ = 0.27 ± 0.06, R^2^ = 0.06 ± 0.03, RMSE = 15.23 ± 0.63), which was not significantly different from the demographic-only model. Shuffled-feature controls for sleep in sleep with demographics, and for brain in all feature sets, reduced performance slightly but not significantly (Fig. 2b, Supplementary Table 4).

Overall, in the Greifswald cohort, demographic variables captured most of the predictive signal for both Stroop and memory test scores, whereas sleep and parcel-based brain morphometry did not significantly improve prediction beyond the demographics-only model.

### Out-of-cohort validation of prediction models

We next tested whether ML models trained in the Greifswald cohort generalized to independent cohorts. We selected AutoGluon as the best-performing model from the Greifswald cohort, which demonstrated robust, stable performance across feature sets. Detailed cohort-level validation results include all metrics for each feature set are provided from Supplementary Table 7 to Supplementary Table 22.

#### Stroop test:

**Figure 3.**
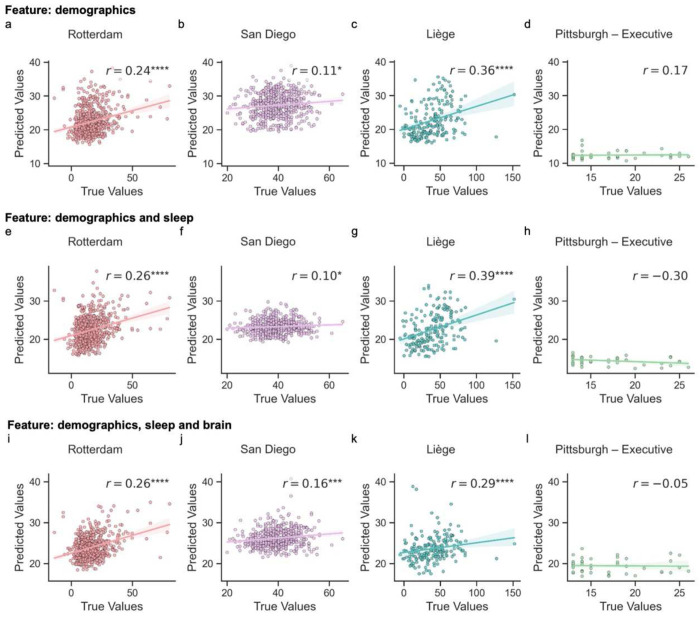
Out-of-cohort validation of Stroop test score prediction by AutoGluon. AutoGluon models were trained in the Greifswald cohort and evaluated on four external cohorts. **a-d,** Model inputs are demographic features only. **e-h,** Model inputs are sleep measurements and demographics. **i-l,** Model inputs are sleep, demographics and parcel-based brain morphometry data. Within each group the panels from left to right correspond to Rotterdam, San Diego, Liège, and Pittsburgh. Each panel displays predicted versus true Stroop test scores. Significance of the Spearman–s rank correlation coefficient is indicated by: *P < 0.05, **P < 0.01, ***P < 0.001, ****P < 0.0001.

Out-of-cohort validation for Stroop test score prediction was assessed in the Rotterdam, San Diego, Liège, and Pittsburgh cohorts. In the Rotterdam cohort, the demographic-only model generalized significantly (Spearman ρ = 0.24, P < 0.0001), the model combining sleep with demographics performed similarly (ρ = 0.26, P < 0.0001), and the all-feature model also generalized (ρ = 0.26, P < 0.0001). In the San Diego cohort, the demographics-only model generalized weakly but significantly (ρ = 0.11, P < 0.05) and the model combining sleep with demographics performed similarly (ρ = 0.10, P < 0.05), whereas the all-feature model showed stronger generalized performance (ρ = 0.16, P < 0.001). In the Liège cohort, the demographics-only model generalized significantly (ρ = 0.36, P < 0.0001), the model combining sleep with demographics generalized significantly (ρ = 0.39, P < 0.0001), and the all-feature model generalized significantly (ρ = 0.29, P < 0.0001). The Pittsburgh cohort contributed an executivefunction proxy rather than a Stroop measure and did not show robust validation results (Fig. 3, Supplementary Table 3). Overall, Stroop test score prediction generalized across Rotterdam, San Diego, and Liège, with the relative performance of the feature sets varying across cohorts.

#### Memory test:

**Figure 4.**
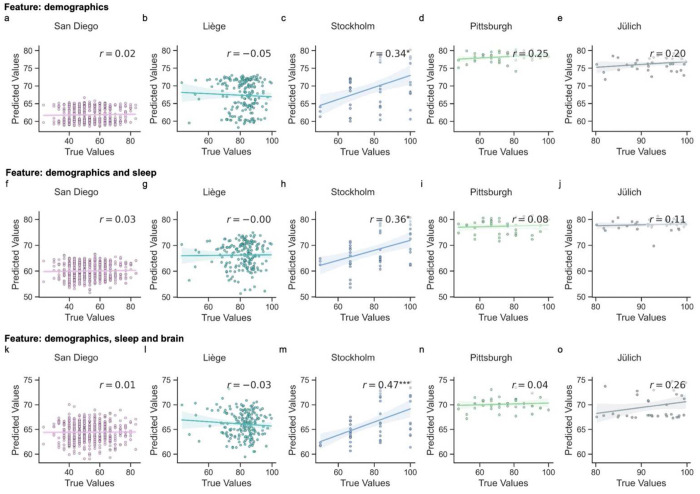
Out-of-cohort validation of memory test score prediction by AutoGluon. AutoGluon models were trained in the Greifswald cohort and evaluated on five external cohorts. **a-e,** Model inputs are demographic features only. **f-j,** Model inputs are sleep measurements and demographics. **k-o,** Model inputs are sleep, demographics and parcel-based brain morphometry data. Within each feature combination, panels from left to right correspond to San Diego, Liège, Stockholm, Pittsburgh, and Jülich. Each panel displays predicted versus true memory test scores. The site-specific memory outcomes were WMS-III Digit Span in San Diego, n-back accuracy in Liège, spatial n-back accuracy in Stockholm, WMS-III Spatial Span in Pittsburgh, and spatial n-back accuracy from session 1 in Jülich. Significance of the Spearman’s rank correlation coefficient is indicated by: *P < 0.05, **P < 0.01, ***P < 0.001, ****P < 0.0001.

Based on data availability, out-of-cohort validation for memory test score prediction was assessed in the San Diego, Liège, Stockholm, Pittsburgh, and Jülich cohorts. The clearest validation was observed in Stockholm, where the demographics-only model generalized significantly (ρ = 0.34, P < 0.05), the model combining sleep with demographics generalized significantly (ρ = 0.36, P < 0.05), and the all-feature model showed the strongest generalized performance (ρ = 0.47, P < 0.001). In contrast, predictions were not validated in the San Diego or Liège cohorts due to differing scales (digit- and letter-based scores), and predictions in the Pittsburgh and Jülich cohorts were modest but not significant (Fig. 4, Supplementary Figure 3, Supplementary Table 4). Overall, out-of-cohort generalizability for memory test score prediction was more limited than for Stroop test score prediction and was most evident in the Stockholm cohort.

### Model explanation in the Greifswald cohort and out-of-cohort validation

To examine how individual features contributed to predictions, we used SHAP values to explain the AutoGluon models trained on the Greifswald cohort. Here, we focused on models that use sleep and demographics because they directly address how sleep measures contribute to prediction while remaining low-dimensional enough for interpretable cross-cohort comparison. SHAP results for models including parcel-based brain morphometry are shown in Supplementary Figures 4 and 5. We focused on models that showed satisfactory external validity, defined a priori as a significant Spearman correlation between observed and predicted scores. Under this criterion, model explanations for predicting the Stroop test score are shown for Greifswald, San Diego, and Liège, and for predicting memory test score for Greifswald and Stockholm.

**Figure 5.**
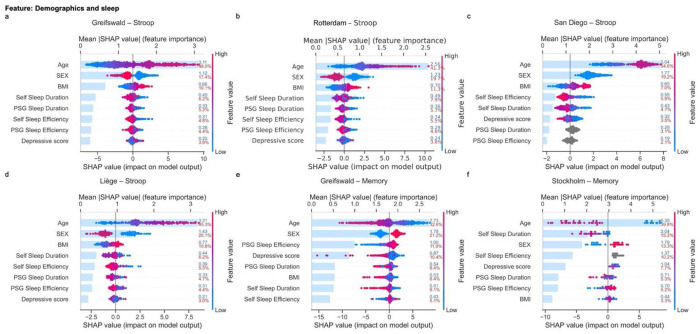
Model explanation by SHAP for Stroop and Memory test score prediction. **a–d**, Model explanations with SHAP for Stroop test score prediction using AutoGluon models with sleep and demographics in Greifswald (a), Rotterdam (b) San Diego (c), and Liège (d). **e–f**, Model explanations for memory test score prediction using the same feature combination in Greifswald (e) and Stockholm (f). Each panel shows a beeswarm plot of SHAP values for individual features, with points representing participants and point color indicating the underlying feature value. Each panel also includes a horizontal bar plot that displays the mean absolute SHAP value of each feature across all participants. Blue numbers next to each bar indicate the mean absolute SHAP value of that feature, and red numbers indicate the percentage contribution of that feature to the total mean absolute SHAP value across all features.

For Stroop test score prediction, age showed the largest mean absolute SHAP values in the sleep and demographics models across all cohorts, followed by sex and BMI. Sleep duration and sleep efficiency contributed only modestly, and depressive symptom scores showed consistently low importance (Fig. 5a–d). In the models including all features, the Greifswald model was dominated by age and 3rd ventricle volume, with additional contributions from prefrontal and cingulate cortical thickness parcels. Overall, cortical thickness features carry more importance than cortical surface area. Similar patterns were observed in the San Diego and Liège cohorts, where age, ventricular volume, and several frontal and cingulate parcels remained among the most important contributors, despite minor site-specific shifts (Supplementary Figure 4).

For memory test score prediction, age again showed the largest mean absolute SHAP values in the sleep and demographic models in both Greifswald and Stockholm. Sex ranked second in Greifswald and third in Stockholm. Sleep measurements contributed more to memory test score prediction than to Stroop test score prediction. In Greifswald, polysomnography-based sleep measurements were more important than self-reported sleep measures, whereas the reverse pattern was observed in Stockholm. BMI showed consistently low importance in predicting memory test scores (Fig. 5e–f). When all features were included, age remained the most important feature in Greifswald, followed by a broader pattern of brain morphometry. Cortical thickness effects were most prominent in the ventromedial and orbitofrontal regions and the occipital cortex, with additional contributions from cortical surface area and ventricular and other subcortical volumes. Sleep variables contributed only marginally. The Stockholm cohort showed a similar pattern, with age again ranking highest and overlapping frontal, occipital, and ventricular markers among the most important features (Supplementary Figure 5).

### Feature interaction and subgroup analysis based on model explanation

We next examined whether the contributions of sleep and demographic variables differed across participants by inspecting pairwise feature interactions and clustering participant-level SHAP profiles from the AutoGluon models trained with sleep and demographic features in the Greifswald cohort. SHAP dependence plots identified age as the dominant feature in both Stroop and memory test score prediction and suggested non-linear interactions between age and sleep duration, with shorter sleep in older participants and longer sleep in younger participants tending to be associated with worse predicted CP (Supplementary Figure 6).

**Figure 6.**
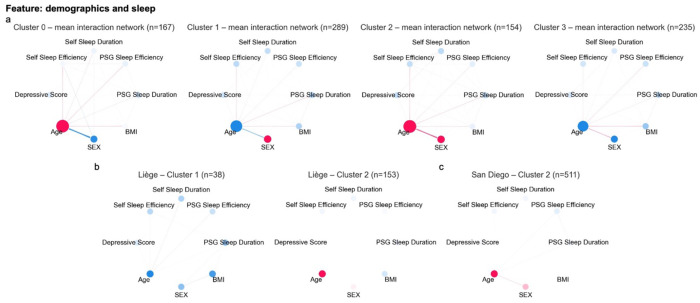
Participants clustering and feature interaction analysis based on SHAP values for Stroop test score prediction. SHAP-IQ interaction networks were computed from AutoGluon models trained with sleep measurements and demographic features for Stroop test score prediction. **a,** Cluster-specific interaction networks for the four SHAP-derived participant subgroups in the Greifswald cohort. **b,** Interaction networks for the two participant subgroups identified when the Greifswald-derived clustering rules were applied to the Liège cohort. **c,** Interaction network for the single participant subgroup identified when the same clustering rules were applied to the San Diego cohort. Node size indicates the mean SHAP value within each subgroup, and node color indicates the direction of the feature contribution to the predicted Stroop test score. Edge thickness and opacity indicate SHAP-IQ interaction strength. For Stroop test scores, higher predicted values indicate lower cognitive performance.

**Figure 7.**
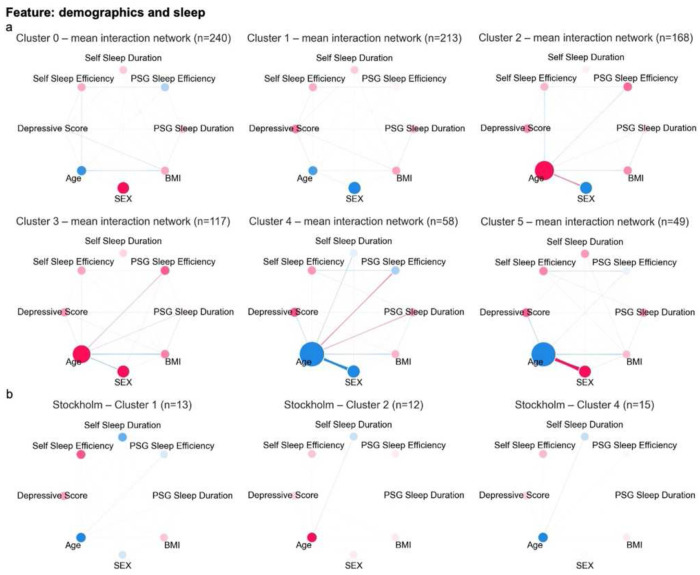
Participants clustering and feature interaction analysis based on SHAP values for memory test score prediction. SHAP-IQ interaction networks were computed from AutoGluon models trained with sleep measurements and demographic features for memory test score prediction. **a,** Cluster-specific interaction networks for the six SHAP-derived participant subgroups in the Greifswald cohort. **b,** Interaction networks for the three participant subgroups identified when the Greifswald-derived clustering rules were applied to the Stockholm cohort. Node size indicates the magnitude of the mean SHAP value within each subgroup, and node color indicates the direction of the feature contribution to the predicted memory test score. Edge thickness and opacity indicate SHAP-IQ interaction strength. For memory test scores, higher predicted values indicate better cognitive performance.

To summarize these heterogeneous explanation patterns, we clustered participant-level SHAP values into subgroups that reflected participants with similar combinations of feature importance for prediction, rather than similarity in the raw input variables. The number of clusters was determined by clustering validity metrics and UMAP visualization (Supplementary Figure 7), yielding four subgroups for Stroop test score prediction (Fig. 6a) and six subgroups for memory test score prediction (Fig. 7a, Supplementary Figure 8). Across these subgroups, age provided the principal axis of separation, whereas the contributions of sleep-related features varied between subgroups. SHAP-IQ further indicated that interactions among age, sex, sleep duration, sleep efficiency, and depressive score were present but generally weaker than the corresponding main effects (Fig. 6a, Fig.7a). We used a decision tree-based algorithm (Goix et al., 2020) to define clustering rules for each subgroup based on the original input values of the AutoGluon model. These rules allowed us to characterize each predicted subgroup profile by identifying the most distinctive variables and value ranges associated with subgroup assignment (Supplementary Tables 5 and 6). Detailed rule-based subgroup definitions, cluster sizes, and cluster-specific interaction patterns are reported in Supplementary Results 1.

When the clustering rules derived in the Greifswald cohort were applied to the external validation cohorts, transferability was cohort-specific, with two Stroop-related subgroup profiles recovered in Liège, one in San Diego (Fig. 6b–c), and three memory-related subgroup profiles in Stockholm (Fig. 7b). The corresponding interaction patterns were broadly similar to those observed in the matched Greifswald subgroups (Supplementary Figures 9 and 10). Supplementary Results 1 further describes which Greifswald-derived subgroup profiles were recovered externally and why incomplete recovery may reflect cohort differences in age distribution, sex composition, sleep measurements, and cognitive measurements.

### Sensitivity analysis based on APOE-ε4

To assess the contribution of APOE-ε4 status to Stroop and memory test score prediction, we trained AutoGluon models that combined APOE with sleep-alone, sleep and demographic, and all features. Then, we compared them to models with shuffled APOE labels and to corresponding models without APOE-ε4 (Supplementary Figure 11). Across all feature sets and outcomes, including APOE-ε4 produced changes in test Spearman ρ that were small in magnitude relative to the corresponding models without APOE-ε4 and did not reach statistical significance in corrected t-tests. Consistent performance across models with observed and shuffled APOE-ε4 indicated that APOE-ε4 status contributed little to predict Stroop and memory test scores in our data.

## DISCUSSION

The present study aimed to predict CP from sets of features (demographic, subjective/objective sleep parameters, and regional brain morphometry) across multisite data. Our findings demonstrated: (i) Non-linear models (i.e., AutoGluon) yielded the most robust performance for both Stroop and memory test score predictions in the largest cohort (Greifswald). Model comparisons across feature combinations further indicated that, unlike demographic-only models, sleep-only features did not provide robust predictive value. Sleep and brain morphometry added limited predictive value to the demographic-only model. Shuffling the sleep and brain features additionally revealed that demographic variables were the primary contributors to prediction performance. In a Greifswald’s subset with available genotype data, adding APOE-ε4 status did not improve cross-validated prediction, indicating limited incremental predictive value in these models; (ii) Out-of-cohort validation of the best-performing predictive model trained on the Greifswald cohort for the Stroop test revealed generalizable patterns across the Rotterdam, San Diego, and Liege cohorts, despite differences in demographic characteristics, including age range, across cohorts. For the memory test, a generalizable pattern was observed only in the Stockholm cohort, highlighting greater variance in acquired memory tests across cohorts; (iii) SHAP-based model explanation showed that age is the main feature contributor for both Stroop and memory test score prediction across all cohorts; (iv) Subgroup analysis based on SHAP values and SHAP-IQ explanations showed heterogeneous patterns for Stroop test score predictions driven by age, sex, and their interaction. For memory test prediction, interactions among age, sex, polysomnography-based sleep efficiency and duration, and depressive score were observed.

Although sleep and brain morphometry are relevant to CP (Kyle et al., 2017; Li et al., 2022), demographic factors, particularly chronological age, emerged as the most robust predictor in our study. This pattern was observed in the primary training dataset and in out-of-sample validation cohorts, suggesting that age captures stable, generalizable variance in CP across independent samples. One possible explanation is that age serves as a proxy for the cumulative effects of biological processes across multiple organs (O’Toole et al., 2026; Tian et al., 2023), many of which are not fully captured by simple measures of sleep duration or regional brain structure. This result is consistent with recent work showing that demographic factors can rival or even outperform multimodal neuroimaging markers in predicting sleep health characteristics and cognitive outcomes, highlighting their strong role in explaining population-level variance (Raimondo et al., 2026; Ujma et al., 2023). Similarly, prior studies have reported that sleep EEG explains only modest variance in cognition and contributes relatively little unique explanatory power after demographic adjustment, typically accounting for approximately 2.5–10% of cognitive variation (Tyurina et al., 2025; Ujma et al., 2023). More broadly, predictive modelling studies in sleep and mental health have shown that neuroimaging can provide limited added predictive utility beyond behavioral measures, clinical variables, and demographic characteristics (Olfati et al., 2024; Raimondo et al., 2026). In population-based studies, the cognitive consequences of poor sleep tend to be modest and heterogeneous (Fjell et al., 2023) and depend on how sleep is operationalized, in terms of duration, efficiency, regularity, fragmentation, or longitudinal change (Moon et al., 2024; West et al., 2024). This heterogeneity likely reflects the inherently dynamic nature of sleep, whose cognitive effects may accumulate over time or depend on chronicity, intra-individual variability, and interactions with aging, affective state, cerebrovascular burden, and circadian regulation (Bi et al., 2026; Tahmasian et al., 2026). A similar interpretation may apply to brain morphometry. Our cross-sectional, parcel-based structural measures likely provide only a coarse anatomical snapshot of neural variation, whereas CP is likely shaped by more distributed and temporally dynamic processes. Although it has been shown that demographic characteristics are also superior to resting-state fMRI in predicting cognitive phenotypes (Omidvarnia et al., 2024). Of note, the amount of our brain features is larger than sleep and demographics. In addition, as a substantial proportion of structural brain variance is age-related, the predictive contribution of morphometric features may be attenuated by age when included in multivariable models (Bi et al., 2026; Komeyer et al., 2025; Kyle et al., 2017). These findings suggest that statistically significant group-level associations between sleep and CP in large-scale UKB data were mainly driven by large sample sizes rather than robust effect magnitudes. This has raised concerns about the practical relevance of the reported small effect sizes in UKB (Keyes & Westreich, 2019; Lin et al., 2013; Spence et al., 2026; Sullivan & Feinn, 2012), and thereby, does not necessarily translate into robust, cross-cohort markers for individual-level prediction, indicating that sleep variables may retain explanatory relevance while offering only limited incremental predictive value.

Our clustering and subgroup analysis were intended to move beyond global SHAP averages and test whether the model relied on the same feature relationships across all participants or instead captured distinct interaction patterns. In this context, an interaction means that part of the model prediction is jointly attributed to a pair of features beyond their separate main effects, so the prediction cannot be fully explained by summing the individual contributions of those features alone. For Stroop prediction, the subgroup patterns indicated that age and sex defined the dominant interaction structure. For example, in older males, age and male sex jointly reinforced a less favorable prediction pattern, whereas in older females, the adverse age effect appeared partly offset by a more favorable sex and age-sex interaction contribution. Thus, even when age remained the dominant feature, its predictive role differed across sex groups rather than following a single, uniform gradient. For memory prediction, the interaction structure was more differentiated and suggested a stronger role for objective sleep within specific demographic contexts. In younger males with better PSG sleep efficiency and lower depressive burden, PSG sleep efficiency and its interaction with age aligned with more favorable predicted memory performance, whereas in older males, objective sleep duration and efficiency appeared to partly buffer an otherwise less favorable age-sex interaction. The repeated divergence between PSG-based and self-reported sleep measures across memory subgroups further suggests that objective and subjective sleep do not contribute interchangeably to prediction. Taken together, these findings suggest that age and sex define the dominant predictive background on which sleep exerts subgroup-specific modifying effects, rather than acting as a uniform predictor across the full sample (Rahimi-Eichi et al., 2025; Sattari et al., 2024; Stankeviciute et al., 2025). Importantly, these clusters should not be interpreted as biological subtypes. Instead, they capture recurring model decision patterns, indicating how demographics and sleep contribute to CP prediction in a heterogeneous and context-dependent manner.

Here, we used ML as it provides an explicit framework for individual-level prediction and for testing generalizability across independent cohorts with different age distributions, which cannot be inferred from within-sample associations alone (Komeyer et al., 2025; Olfati et al., 2024; Omidvarnia et al., 2024; Rosenblatt et al., 2026; Wu et al., 2023). This design allowed us to test whether models trained in the broad adult age range of the Greifswald cohort retained predictive performance in cohorts with different age ranges (Mendl-Heinisch et al., 2026; Olfati et al., 2024), while recognizing that transferability remained outcome and cohort-dependent. Recent studies in sleep and mental health underscore the utility of predictive modeling, enabling validation across independent datasets and quantification of the incremental value of candidate feature domains (Olfati et al., 2024). Importantly, recent large-scale neuroimaging studies further illustrate that ML can serve as a stringent test of whether high-dimensional brain features meaningfully improve out-of-sample prediction. In some cases, predictive performance remains near chance, thereby placing important constraints on mechanistic interpretation and tempering expectations for clinical translation (Olfati et al., 2024; Omidvarnia et al., 2024; Raimondo et al., 2026). Although many AI applications in sleep medicine focus on diagnostics, staging, or monitoring rather than cognitive performance itself, they highlight a methodological point that is directly relevant here, namely that real-world utility depends on rigorous validation, transparent reporting, and explicit handling of heterogeneity and bias (Bi et al., 2026; Jahrami et al., 2026). These considerations motivated our unified, leakage-controlled ML framework across multiple cohorts. By strictly separating model development from external evaluation and fitting all preprocessing steps within each training split, the analysis yields conservative estimates of generalization under realistic site heterogeneity. Finally, SHAP and SHAP-IQ added an important explanatory layer by distinguishing dominant global effects from smaller context-dependent effects and by revealing subgroup-specific interaction patterns that were not evident from performance metrics alone.

Our study has several limitations, which mainly concern the existing predictor domains, the comparability of cognitive targets across cohorts, and the methodological constraints of cross-cohort ML validation (Bi et al., 2026; Raimondo et al., 2026). First, we only focused on sleep duration and sleep efficiency from PSG and the Pittsburgh Sleep Quality Index (PSQI), which represent a subset of sleep health dimensions. Future work should incorporate long-term wearable-derived measures to capture night-to-night variability at scale (Massar et al., 2021). Multimodal sleep data, including detailed biological signals and subjective reports, and emerging sleep-focused foundation models may further improve representation of learning across cohorts under rigorous train-test separation (Thapa et al., 2026). Second, the cognitive phenotypes showed limited comparability across cohorts and likely variable reliability, particularly for memory, which may have limited out-of-cohort prediction and reduced apparent generalizability across sites (Gell et al., 2024; Rosenblatt et al., 2026). This may be more salient in multicohort settings where CP measures differ in content and scaling, where harmonization supports valid external validation. A recent proposed framework removes site-related variance while preserving instrument effects and links tests via a shared latent ability scale to facilitate cross-cohort score conversion (Kennedy et al., 2024, 2025). However, it relies on item-level data and is less applicable when only total scores are available. In ML settings, harmonization may yield optimistic estimates if information from hold-out participants influences the transformations, which motivates leakage-free harmonization that fits only the training data (Nieto et al., 2026). As the included cohorts differed in age distributions (Fig. 1b), depressive scores (Supplementary Table 1), and implementation of the cognitive tasks, we did not harmonize features or targets across sites to avoid cross-site information leakage (Nieto et al., 2026). Longitudinal designs will be essential to determine whether sleep and multimodal brain measures (e.g., white matter integrity, functional connectivity, vascular burden, or brain age gap) predict future cognitive decline more strongly than baseline CP.

## CONCLUSION

This study suggests that while sleep and brain structure make modest contributions in predicting CP, age remains the primary and generalizable predictor in our multisite, individual-level models. Overall, these results indicate a complex contribution of demographic characteristics, sleep, and brain structure to CP. Our findings underscore the need for longitudinal, multimodal, and densely sampled data, as well as personalized approaches, to better understand how aging, sleep, and brain structure jointly shape CP at the individual level.

## METHODS

### Study participants and measurements

#### Study participants:

Participants were included from seven cohorts of the ENIGMA-Sleep Working Group, yielding a total of 2,372 participants (N = 938 females; median age = 58.8 years; range = 18.0 – 95.1 years). Details of the cohorts’ demographic characteristics, depressive scores, APOE-ε4 carrier status, and sleep measurements are summarized in Supplementary Table 1. All contributing sites received approval from their respective local institutional review boards and ethics committees to collect and analyze data. Cohort-specific inclusion and exclusion criteria before data sharing are listed in Supplementary Table 2.

#### Sleep measurements:

Sleep duration and sleep efficiency were included as sleep predictors. These measures were derived from PSG and self-reported sleep questionnaires where available. For questionnaire-based sleep measures, the PSQI was used across cohorts with self-reported sleep data, and the Karolinska Sleep Questionnaire (KSQ) (Nordin et al., 2013) was used only in the Stockholm cohort. The availability and modality of sleep measures differed across cohorts and are summarized in Supplementary Table 1. We retained both objective and self-reported measures when both were available, because they capture related but non-equivalent aspects of sleep.

#### Demographic variables:

Age, sex, body mass index (BMI), and depressive scores were included, where available, as the demographic and clinical feature set used for prediction. Age was included because cognitive performance, sleep, and brain morphometry vary across adulthood. Sex was included because it is relevant to inter-individual differences in sleep, brain structure, and cognition. BMI was included because it is associated with sleep and overall health status, which may affect cognitive performance. Depressive scores were included because depressive symptoms are associated with both sleep and cognition. These variables were entered as predictors and were not regressed out from the input features or cognitive outcomes. Depressive symptoms were assessed with the Patient Health Questionnaire-9 (PHQ-9) in Greifswald, the Beck Depression Inventory-II (BDI-II) (Beck et al., 2011) in Liège, the Center for Epidemiologic Studies Depression Scale (CES-D) (Radloff, 1977) in Rotterdam and San Diego, and the Hospital Anxiety and Depression Scale (HADS) (Zigmond & Snaith, 1983) in Stockholm. PHQ-9 was converted to the Beck Depression Inventory-II (BDI-II) scale before analysis. HADS scores in Stockholm were transformed to the BDI-II scale using a piecewise mapping based on severity ranges, with HADS scores of 0–7 mapped to BDI-II 0–13, 8–10 to 14–19, 11–15 to 20–28, and 16–21 to 29–63, followed by rounding to the nearest integer. CES-D scores were retained on their original scale. Depressive scores were not available in Pittsburgh or Jülich.

#### Stroop test score:

The Stroop test score was used as an executive function-related outcome. It was measured as reaction time, defined as the interference cost in seconds computed as the difference between the interference and neutral conditions in the Greifswald, Rotterdam, and Liège cohorts. In the San Diego cohort, the Stroop test score was indexed by the Stroop Interference Norm-Based T-Score (Golden et al., 1978), which reflects performance on the color-word interference condition. The Pittsburgh cohort did not provide a Stroop measure and contributed an executive function proxy using the Neuropsychological Assessment Battery (NAB) Mazes Test. To ensure consistency across cohorts, in which lower Stroop test scores indicate better cognitive performance, we inverted the San Diego and Pittsburgh scores by subtracting each score from the sum of the cohort-specific maximum and minimum. Although measurement protocols differed across cohorts, the scales of these Stroop test scores were comparable (Supplementary Figure 1), and models were therefore trained in the Greifswald cohort and evaluated on other independent cohorts to assess the generalizability of ML models.

#### Memory test score:

The memory test score was used as an accuracy-based memory outcome. It reflected accuracy for n-back working memory tasks in the Liège, Stockholm, and Jülich cohorts. In the Liège cohort, working memory performance was measured using the n-back paradigm with three load conditions: 1-, 2-, and 3-back. To unify this measure with accuracy-based memory scores from other cohorts, we computed one memory test score per participant by taking the mean accuracy across the three n-back loads. In the Jülich cohort, working memory was assessed with letter and spatial versions of an n-back task in which participants indicated whether the current stimulus matched the one presented N trials earlier. For each load level (1-, 2-, and 3-back), performance was summarized by the sensitivity (A’) based on hits, false alarms, and omissions (Hennecke et al., 2021). We derived a single working memory score by averaging A’ across the available n-back loads, including both letter and spatial versions.

In Stockholm, a spatial n-back paradigm was used, implemented in the WakeApp web app (Holding et al., 2021). Participants were presented with a grid in which squares light up in sequence, and the task was to remember the sequence. After each sequence, one square lit up with a number to ask whether that square was in the position indicated by the number in the sequence, and the participant responded yes or no. The test included 6 6-long sequences and 6 2-long sequences.

In the Greifswald cohort, memory was assessed using the Nuremberg Age Inventory (NAI) word list (Oswald & Fleischmann, 1999), a German verbal learning and memory test that presents eight words and requires immediate and delayed recall in the presence of distractor words. Immediate recall was scored as the number of correctly recalled target words, and delayed recognition was scored as the number of correctly recognized target words minus the number of falsely endorsed distractor words, with a maximum of eight points for each condition. In the present study, we derived a memory accuracy score by dividing the immediate and delayed sum scores by their maximum possible value and averaging the two scaled scores.

In the San Diego cohort, memory performance was assessed using the Wechsler Memory Scale–Third Edition (WMS-III) Digit Span Forward, Digit Span Backward, and Letter-Number Sequencing (Wechsler, 1997). For Digit Span, we summed the forward and backward raw scores and divided the sum by the maximum possible score of 30 to obtain a digit span accuracy proportion. For Letter Number Sequencing, we divided the total raw score by the maximum possible score of 21 to obtain a sequencing accuracy proportion.

The Pittsburgh cohort contributed memory measures from the WMS-III Spatial Span and Letter-Number Sequencing. Spatial Span Forward and Backward raw scores were combined into a total score between 0 and 32. Letter-Number Sequencing yielded a total raw score ranging from 0 to 21. To place these measures on a common accuracy scale, we transformed each raw score into a percentage score by dividing the Spatial Span total by its maximum of 32 and the Letter-Number Sequencing total by its maximum of 21.

#### APOE-ε4 carriership:

APOE-ε4 carriership: APOE-ε4 carrier status was summarized where available and used in sensitivity analyses to explore whether genetic risk information improved the prediction of Stroop and memory test scores beyond the main feature sets. In San Diego data, APOE genotype was determined using PCR and HhaI restriction digest methods. In Greifswald, Rotterdam, Liège, and Stockholm, the APOE genotype was determined from imputed rs429358 and rs7412 variants. APOE-ε4 carriership by cohort is reported in Supplementary Table 1.

### Image acquisition and processing

Each cohort collected T1-weighted structural MRI at participating sites. Images were processed in each cohort by the standard ENIGMA workflow with FreeSurfer (https://github.com/ENIGMA-git/). Cohort-specific image acquisition parameters and FreeSurfer version are included in the Supplementary Table 2. For each participant, parcel-based cortical thickness and surface area were calculated using the Schaefer atlas (400 regions and 7 networks) (Schaefer et al., 2018). Parcel-based subcortical volumes were calculated using the ASEG atlas, yielding 36 volumetric regions, including bilateral lateral ventricles, inferior lateral ventricles, cerebellar white matter, cerebellar cortex, thalamus, caudate, putamen, pallidum, hippocampus, amygdala, nucleus accumbens, ventral diencephalon, and vessels, as well as the third, fourth, and fifth ventricles, cerebrospinal fluid, brainstem, and five corpus callosum segments (Fischl et al., 2002).

### Feature integration and quality control

For each cohort, demographic information, sleep measurements, and parcel-based brain morphometry features were combined into a tabular dataset with one row per participant. Participants with missing phenotypic or imaging features were excluded. To detect potential image-processing failures and extreme multivariate outliers, we applied Isolation Forest (Liu et al., 2008) implemented by Scikit-learn (Pedregosa et al., 2011) within each cohort. The Isolation Forest was fitted on all features and assigned each participant an inlier (+1) or outlier (−1) label, and participants classified as outliers were excluded. This procedure was used only to detect and exclude participants, and did not modify feature values, use outcome information, or create any derived dataset for subsequent analysis. This step was treated as cohort-level quality control before model training rather than as a cross-validation preprocessing transformation. It was performed without using Stroop or memory test scores, and the resulting outlier labels were not estimated from, or optimized against, prediction performance.

We did not apply cross-site data harmonization in this study because standard harmonization methods such as ComBat (Johnson et al., 2007) and ComBat-GAM (Pomponio et al., 2020) can introduce data leakage in ML pipelines when they are fitted across combined training and test data or across cohorts before cross-validation (Nieto et al., 2026). In addition, previous work has suggested that appropriately trained ML models can mitigate site and demographic-related bias in performance without explicit harmonization (Wang et al., 2023).

### Machine learning models

To comprehensively characterize the link between sleep, demographics, brain morphometry, and cognitive performance, and to assess both linear and nonlinear relationships, we benchmarked a panel of seven supervised learning algorithms. These ranged from simple linear models, which are linear regression, ridge regression, and SVM with linear kernel, to nonlinear kernel and tree-based ensembles, which are SVM with rbf kernel, random forest, and XGBoost, and an automated ML framework i.e., AutoGluon. AutoGluon, as an automated tabular learning framework, can infer feature types, handle common preprocessing steps such as missing data and feature rescaling, and train a diverse set of base learners that are combined through bagging, stacking, and a greedy weighted ensemble. This spectrum of models also allowed us to evaluate whether associations between sleep, brain morphometry, and cognition were better captured by models that can represent nonlinear relations (Li et al., 2022) and whether these associations were robust across different algorithm choices.

### Model training and internal evaluation

Model training took place in the Greifswald cohort, which had the largest sample size, covered the age range of the other cohorts, and included both Stroop and memory test scores. Prior to model training, parcel-based morphometric features were ratio-adjusted for brain size to mitigate global scaling effects (Nicolaisen-Sobesky et al., 2022). Depressive scores were entered as model predictors within the demographics and were not used for residualizing either predictors or cognitive outcomes. Specifically, cortical thickness, surface area, and subcortical volume were normalized by overall cortical thickness, total surface area, and total intracranial volume (TIV) at the individual participant level. To ensure generalizability and obtain test scores from the Greifswald cohort, we employed a 5-fold, 10-repeat nested cross-validation (CV), yielding 50 outer train-test splits for model evaluation. To maintain distributional consistency across folds, data splitting was stratified based on a demographic label generated by binning age into tertiles (based on the Greifswald cohort quantiles) and interacting these age bins with sex.

For linear regression, ridge regression, SVM with linear kernel, SVM with rbf kernel, random forest, and XGBoost models, feature preprocessing and hyperparameter tuning were conducted within the training data of each outer split. For each outer split, continuous variables in the training data were standardized with z-scores. The fitted transformers were then applied to the corresponding test data for that split. Within each outer training set, hyperparameters for linear regression, ridge regression, SVM with linear and rbf kernels, and random forest were tuned by grid search using 5-fold inner CV with coefficient of determination (R^2^) as the optimization metric, implemented with Julearn (Hamdan et al., 2024) and Scikit-learn (Pedregosa et al., 2011). For XGBoost, Optuna optimized hyperparameters using 5-fold inner CV with R^2^ as the optimization metric (Akiba et al., 2019). The best hyperparameters from each outer split were used to refit the model on that split’s full training data, and performance was then evaluated on that split’s test data.

For AutoGluon models, in each outer split, models were trained on the original feature values in the training data, since AutoGluon includes internal logic for preprocessing operations such as missing value imputation and one-hot encoding. We used AutoGluon’s TabularPredictor in regression mode with R^2^ as the optimization metric and the best-quality preset, which trains a diverse stack ensemble of bagged models and automatically configures hyperparameters, stacking and bagging based on dataset properties (Erickson et al., 2020). In each outer split, the single best-performing model obtained from the training procedure was refitted on the training data and evaluated on that split’s test data.

We summarized performance across outer splits by averaging each test metric from each split to obtain model performance in the Greifswald cohort. Spearman correlation between observed and predicted scores was the primary performance metric because it evaluates whether participants with higher observed cognitive performance also tended to receive higher predicted scores, is less sensitive to extreme values than Pearson correlation, and is less dependent on absolute score scaling across different cognitive tests. We additionally reported rooted mean squared error (RMSE), mean absolute error (MAE), R^2^, and Pearson correlation. Linear regression, ridge regression, SVM with linear and rbf kernel, random forest, and XGBoost were then refitted with the best performing hyperparameters on the full Greifswald dataset for out-of-cohort validation. AutoGluon models were retrained on the whole Greifswald cohort using the same configuration as in nested CV for out-of-cohort validation.

### Model comparison on the Greifswald cohort

To evaluate the contribution of different feature sets to prediction, we trained models for each ML algorithm on the Stroop test score and the memory test score using multiple input feature sets, which comprised sleep measurements only, demographics only, sleep with demographics, parcel-based brain morphometry only, and sleep plus demographics with brain. In addition, as negative control models, we trained models in which sleep measurements were randomly shuffled while demographics and brain morphometry features were left intact, and models in which brain morphometry features were randomly shuffled while sleep and demographics were left intact. These negative control models were used to assess whether sleep or brain features added information beyond demographics.

Statistical comparisons of model performance were carried out within the Greifswald cohort to compare feature sets within each algorithm and to compare algorithms within each feature set. Comparisons were based on model performance on the outer split and used the Nadeau–Bengio corrected resampled paired t-test on nested cross-validation scores with Bonferroni adjustment for all pairwise contrasts with threshold (Nadeau & Bengio, 1999). As all algorithms used the same outer fold splits, these pairwise comparisons were comparable. Statistical comparisons were implemented with Julearn (Hamdan et al., 2024).

### Out-of-cohort validation in independent cohorts

For out-of-cohort validation, models were trained in the Greifswald cohort and then applied separately to the Rotterdam, San Diego, Liège, and Pittsburgh cohorts for Stroop test score prediction, and to the San Diego, Liège, Stockholm, Pittsburgh, and Jülich cohorts for memory test score prediction. Consistent with model training and evaluation in the Greifswald cohort, cortical thickness, cortical surface area, and subcortical volumes were normalized by overall cortical thickness, total cortical surface area, and total intracranial volume at the individual participant level in each validation cohort. Based on the algorithm comparison in the Greifswald cohort, we focused on out-of-cohort validation on XGBoost and AutoGluon, which showed robust and stable performance across feature sets. For XGBoost, the z-score preprocessing parameters estimated from the full Greifswald dataset were applied to the corresponding features in each validation cohort. For each feature set, the final model refitted in the Greifswald cohort was then applied to each validation cohort. AutoGluon models retrained in the Greifswald cohort for each feature set were applied directly to the validation cohorts. Spearman correlation between observed and predicted scores was the primary performance metric, and we additionally reported RMSE, MAE, R^2^, and Pearson correlation. For both Spearman and Pearson correlation, we computed two-sided p-values to assess whether predictions were significant.

### Model explanation

The contribution of different feature sets to prediction was assessed by model comparison, whereas the contribution of individual features within a model was quantified with SHAP values. SHAP values were computed with the SHAP toolbox (Lundberg & Lee, 2017). Model explanations focused on AutoGluon, which showed stable and comparatively strong predictions across feature sets. For each prediction target and feature set, we used the AutoGluon model retrained on the full Greifswald cohort and applied the SHAP PartitionExplainer, which better handles correlations among features, particularly for parcel-based brain features, using the full Greifswald dataset as background data to derive SHAP values for all participants in Greifswald. The same explainer was then applied to out-of-validation cohorts by passing the corresponding cohort data as new input while keeping the background data and AutoGluon model fixed.

AutoGluon models trained with all feature sets were explained in the Greifswald cohort. For reporting, we focused on two feature sets. First, sleep with demographics, which addresses how sleep measures and demographics contribute to prediction without imaging features. Second, all features, including sleep, demographics, and brain morphometry, show individual feature contributions when all features are available. Out-of-cohort SHAP analyses were restricted to cohorts in which the AutoGluon model achieved statistically significant out-of-cohort validation performance, defined as a significant Spearman correlation between observed and predicted scores (two-sided P < 0.05). We therefore report model explanations for the San Diego and Liège cohorts in Stroop prediction and for the Stockholm cohort in memory prediction to avoid overinterpreting feature attributions in cohorts with low prediction performance.

Global feature importance was summarized as the mean absolute SHAP value per feature and visualized with bar plots. Local feature attributions were represented by the distribution of SHAP values across participants and visualized with beeswarm plots. For Stroop test score prediction, higher SHAP values indicate that a feature contribution pushes the model towards larger predicted Stroop test scores, corresponding to worse executive performance. For memory test score prediction, higher SHAP values indicate that a feature contribution pushes the model towards larger predicted memory scores, corresponding to better memory performance.

### Feature interaction analysis based on model explanation

SHAP dependence plots were used to qualitatively visualize how feature values are related to their contributions to model predictions and to suggest potential feature interactions. Because conventional SHAP values assign prediction deviations to individual features and may distribute interaction effects across single-feature attributions, they do not directly quantify pairwise interactions. To quantify pairwise interaction effects beyond the marginal feature attributions provided by SHAP, we used the Shapley Interaction Quantification (SHAP-IQ) toolbox (Muschalik et al., 2024). Similar to the SHAP analysis, we applied the SHAP-IQ TabularExplainer to the AutoGluon models retrained on the full Greifswald cohort, using the full Greifswald dataset as background data, and derived SHAP values and pairwise interaction values for all participants in Greifswald. The same explainer was then applied to out-of-validation cohorts by passing the corresponding cohort data as new input while keeping the background data and model fixed. Out-of-cohort SHAP-IQ analyses were restricted to cohorts in which the AutoGluon model achieved satisfactory external validation performance, which are the San Diego and Liège cohorts for Stroop prediction and the Stockholm cohort for memory prediction. The construction and interpretation of interaction networks based on these quantities is described in the subgroup analysis section.

Feature interactions analysis, derived from model explanations focused on sleep, with the demographic feature set. We restricted detailed interaction analyses to sleep with demographics, as this feature set directly targeted how sleep metrics interact with demographic factors in predicting cognitive performance and remained sufficiently low-dimensional for stable and interpretable interaction estimates. Including parcel-based brain morphometry features would have created a very high-dimensional interaction space with many weak, correlated effects.

### SHAP-based subgroup analysis

To quantitatively characterize potential feature interactions that differed across participant groups, as suggested by the SHAP dependency plots, we clustered participant-level SHAP values to characterize potential feature interaction patterns that differed across subgroups. Subgroups were derived from SHAP values obtained from AutoGluon models trained on sleep and demographic features to predict Stroop and memory test scores in the Greifswald cohort. For each participant, we constructed an input vector consisting of SHAP values for all sleep and demographic features and used these SHAP vectors as the space for clustering. As different clustering algorithms impose different geometric and probabilistic assumptions on the data, we evaluated spectral clustering, k-means, and Gaussian mixture models. Algorithm choice and the number of clusters were guided by the gap statistic, silhouette score, Bayesian information criterion, and an elbow analysis of within-cluster sum of squares (Schubert, 2023), together with visual inspection of the resulting clustering structure. The final solution was selected as the combination of algorithm and cluster number that yielded clearly separated and interpretable subgroups. Uniform Manifold Approximation and Projection (UMAP) was used to embed the SHAP vectors into two dimensions for visualization (McInnes et al., 2020). Clustering algorithms and cluster validity metrics were implemented with scikit-learn, and UMAP was implemented with the Python UMAP package.

Each subgroup’s model explanations were visualized using SHAP beeswarm plots and SHAP-IQ interaction networks. The beeswarm plots reveal which features dominate the predictions within a subgroup and how the directions and magnitudes of their effects vary across individuals, whereas the SHAP-IQ interaction networks expose subgroup-specific patterns of interacting features. For each subgroup’s SHAP-IQ interaction network, we aggregated individual SHAP-IQ interaction values by averaging all participants in that cluster to obtain a subgroup-level interaction pattern.

To obtain human-readable cluster descriptions, we used SkopeRules (Goix et al., 2020) to learn decision rules that predicted SHAP-derived cluster labels from the original AutoGluon training features. We trained SkopeRules in the original feature space rather than on SHAP values because the raw variables are directly interpretable and consistently defined across cohorts. To transfer subgroup assignments to out-of-validation cohorts, the rules learned in the Greifswald cohort were applied directly as feature conditions. Participants were assigned to a subgroup when exactly one rule was satisfied and were left unassigned when no rule or more than one rule matched. For each subgroup in the out-of-validation cohorts, model explanations were also visualized using SHAP beeswarm plots and SHAP-IQ interaction networks.

### Sensitivity analyses

Given its established relevance to cognitive performance and dementia, we conducted a sensitivity analysis to assess whether adding APOE-ε4 status improved prediction of Stroop and memory test scores from the other features. APOE-ε4 carrier status was available for a subset of participants (N = 785) in the Greifswald cohort. Within this cohort, we repeated the AutoGluon training and evaluation for Stroop and memory test score prediction using the same 5-fold 10-repeat nested CV design and evaluation metrics as in the main AutoGluon models. Because APOE-ε4 did not improve cross-validated prediction within the Greifswald subset, APOE-based models were not advanced to out-of-cohort validation.

For both Stroop and memory test score prediction, we considered three feature configurations based on the main AutoGluon models, that is, sleep-only, sleep plus demographic, and all features. For each configuration, we trained two additional variants of the AutoGluon model: one model including APOE-ε4 as an additional feature and another negative control model, including a randomly shuffled APOE-ε4 feature. As in the main analyses, performance of the APOE-ε4 models was summarized by the same set of metrics, with Spearman correlation as the primary metric and MSE, MAE, R^2^, and Pearson correlation also reported, averaged across outer test folds. To quantify the incremental predictive value of APOE-ε4, we compared model performance between models with and without APOE-ε4 and between models with observed and permuted APOE-ε4 status using Nadeau–Bengio corrected resampled paired t-tests with Bonferroni adjustment for all pairwise contrasts.

## Supplementary Material

This is a list of supplementary files associated with this preprint. Click to download.

- SupplementaryMaterials.pdf

## Data Availability

The data used in this study were collected independently at participating sites and analyzed following the standardized ENIGMA protocols. Due to participant privacy considerations, ethical restrictions, and site-level data-sharing agreements, individual-level data cannot be made publicly available. Data requests will be reviewed by the respective site and must comply with local ethics approvals and data-use agreements. For the Greifswald SHIP-Trend cohort, data access applications should be submitted through the SHIP transfer portal at https://transfer.ship-med.uni-greifswald.de/FAIRequest/?lang=en and will be reviewed by the SHIP committee. For the San Diego cohort, instructions for data access requests are available on the Vietnam Era Twin Study of Aging (VETSA) website at https://psychiatry.ucsd.edu/research/programs-centers/vetsa/researchers.html. De-identified VETSA Wave 1, 2, and 3 data are publicly available through the National Archive of Computerized Data on Aging at https://www.icpsr.umich.edu/web/ICPSR/studies/38836. For the Stockholm cohort, anonymized data are available at https://openneuro.org/datasets/ds000201.

## Consortia

### ENIGMA Sleep Working Group

Hanwen Bi, Michele Deantoni, David Elmenhorst, Fabio Ferrarelli, Hans J. Grabe, Sanne J.W. Hoepel, Felix Hoffstaedter, Neda Jahanshad, Ahmad Mayeli, Nasrin Mortazavi, Julia Neitzel, Gustav Nilsonne, Amin Saberi, Christina Schmidt, Kai Spiegelhalder , Sandra Tamm, Sophia I. Thomopoulos, Paul M. Thompson, Sofie L. Valk, Gilles Vandewalle, Henry Völzke, Joseph Wexler, Kaustubh R. Patil, Antoine Weihs, Simon B. Eickhoff, Federico Raimondo, Masoud Tahmasian

## Code availability

This study was preregistered on the Open Science Framework (OSF, https://osf.io/nhbkq/). All analysis tools are open-source in this study based on Python 3.10.18 using NumPy 2.1.3, pandas 2.3.3, SciPy 1.15.3, scikit-learn 1.7.2, XGBoost 3.0.5, AutoGluon 1.2.0, SHAP 0.49.1, SHAP-IQ 1.3.1, Skope-Rules 1.0.1, and UMAP-learn 0.5.7. Brain visualizations were generated based on Python 3.11.14 with NumPy 1.26.4, nilearn 0.12.1, nibabel 5.3.2, neuromaps 0.0.5, brainspace 0.1.20, and surfplot 0.2.0. All scripts for analyses in this study and Python environment specifications are provided in the GitHub repository (https://github.com/harveybi/ENIGMA-Sleep-Cognitive-Performance).
